# Risk of Suboptimal Iodine Intake in Pregnant Norwegian Women

**DOI:** 10.3390/nu5020424

**Published:** 2013-02-06

**Authors:** Anne Lise Brantsæter, Marianne Hope Abel, Margaretha Haugen, Helle Margrete Meltzer

**Affiliations:** 1 Department of Exposure and Risk Assessment, Division of Environmental Medicine, Norwegian Institute of Public Health, P.O. Box 4404, Nydalen, NO-0403, Norway; E-Mails: Margaretha.Haugen@fhi.no (M.H.); Helle.Margrete.Meltzer@fhi.no (H.M.M.); 2 Tine SA Oslo Norway, P.O. Box 25, 0051 Oslo, Norway; E-Mail: Marianne.Hope.Abel@tine.no

**Keywords:** iodine, pregnancy, prospective cohort, food frequency questionnaire, the Norwegian Mother and Child Cohort Study (MoBa)

## Abstract

Pregnant women and infants are exceptionally vulnerable to iodine deficiency. The aims of the present study were to estimate iodine intake, to investigate sources of iodine, to identify predictors of low or suboptimal iodine intake (defined as intakes below 100 μg/day and 150 μg/day) in a large population of pregnant Norwegian women and to evaluate iodine status in a sub-population. Iodine intake was calculated based on a validated Food Frequency Questionnaire in the Norwegian Mother and Child Cohort. The median iodine intake was 141 μg/day from food and 166 μg/day from food and supplements. Use of iodine-containing supplements was reported by 31.6%. The main source of iodine from food was dairy products, contributing 67% and 43% in non-supplement and iodine-supplement users, respectively. Of 61,904 women, 16.1% had iodine intake below 100 μg/day, 42.0% had iodine intake below 150 μg/day and only 21.7% reached the WHO/UNICEF/ICCIDD recommendation of 250 μg/day. Dietary behaviors associated with increased risk of low and suboptimal iodine intake were: no use of iodine-containing supplements and low intake of milk/yogurt, seafood and eggs. The median urinary iodine concentration measured in 119 participants (69 μg/L) confirmed insufficient iodine intake. Public health strategies are needed to improve and secure the iodine status of pregnant women in Norway.

## 1. Introduction

Iodine is an essential component of the thyroid hormones. It is required throughout the life-cycle and is obtained primarily through the diet. Iodine deficiency is the world’s greatest single cause of preventable brain damage, and in the last few decades, there has been remarkable progress in the global effort to eliminate iodine deficiency [1,2,3]. Insufficient iodine status is not only a problem in developing countries, but is also characterized as a major public health problem in many countries in Europe and the Western world [1,4,5,6].

Before 1950, there was endemic iodine deficiency in Norway, with goiter prevalence as high as 80% in certain inland areas [7]. Since then, iodine has been added to cow fodder, resulting in a relatively high concentration of iodine in milk and dairy products. Combined with a high consumption of milk and other dairy products, this led to eradication of endemic goiter [8]. The same happened in Britain [9], while other countries introduced national iodization programs by adding iodine to salt [5,10].

Pregnant women and infants are exceptionally vulnerable to deficiency, because iodine is essential for normal fetal and child brain development and growth [4,11]. The estimated average requirement for iodine in non-pregnant adults is 95 μg/day, and the recommended intake of 150 μg/day is derived by adding 2 SD and rounding to the nearest 50 μg/day [3,5]. The estimated average requirement is increased by at least 50% during pregnancy and lactation [3,12]. The recommended iodine intake during pregnancy is 175 μg/day in Nordic countries [13], while WHO/UNICEF/ICCIDD recommends 250 μg/day during pregnancy and lactation [14]. According to the epidemiological criteria for assessment of iodine status in pregnant populations, a median urinary iodine concentration (UIC) below 150 μg/L defines iodine deficiency (insufficient iodine intake), while a median UIC in the range 150–249 μg/L defines adequate (optimal) iodine intake [3,14,15]. Iodine deficiency during pregnancy is of concern when iodine intake falls below 100 μg/day [16].

Iodine deficiency has not been considered an issue in developed countries, such as Norway, for many decades. However, a sub-study in 119 pregnant women recruited to the Norwegian Mother and Child Cohort study (MoBa) in 2003–2004 revealed low iodine intake [17,18]. Low iodine intake was also seen in a study within a larger sample of MoBa, with 10% of the women having iodine intake below 70 μg/day [19]. Studies on iodine intake and iodine status of pregnant women are urgently needed.

There have been major changes in Norwegian food patterns over the last decades. The aims of the present study were to estimate iodine intake, to investigate sources of iodine, to identify predictors of low or suboptimal iodine intake (defined as intakes below 100 μg/day and 150 μg/day, respectively) in a large population of pregnant Norwegian women and to evaluate iodine status in a sub-population of the participants.

## 2. Materials and Methods

### 2.1. Population and Study Sample

The data set is part of the Norwegian Mother and Child Cohort study (MoBa), a prospective population-based pregnancy cohort conducted by the Norwegian Institute of Public Health [20]. Participants were recruited from all over Norway from 1999 to 2008, and 38.5% of invited women consented to participate. The cohort now includes 108,000 children, 90,700 mothers and 71,500 fathers. Blood samples were obtained from both parents during pregnancy and from mothers and children (umbilical cord) at birth. Follow-up is conducted by questionnaires at regular intervals and by linkage to national health registries. Several sub-studies are conducting additional collections of data and biological materials.

The data included in this study are from two questionnaires answered in gestational weeks 15 (Q1) and 17–22 (Q2), respectively. Q2 is a detailed food frequency questionnaire (FFQ), while Q1 is a general questionnaire covering health, exposures, lifestyles and background factors. Pregnancy and birth records from the Medical Birth Registry of Norway (MBRN) are linked to the MoBa database [21]. Informed consent was obtained from each participant before the study. The study was approved by the Regional Committee for Ethics in Medical Research and the Data Inspectorate in Norway.

This study uses the quality-assured data files released for research in 2009 (version 4). At the time of this analysis, 89,656 women had answered the first MoBa questionnaire and were recorded in MBRN. Of these, 76,218 had also answered version 2 of the FFQ [22], and 74,914 had registered a valid food intake (total energy >4500 kJ and <20,000 kJ per day). The range of acceptable energy intake in MoBa has been evaluated elsewhere [22]. We excluded 5447 participants with missing information on maternal height, weight, smoking habits, educational attainment or household income and 7563 women who had participated in MoBa with more than one pregnancy, resulting in a study sample of 61,904 women for analysis. 

Finally, we included data from a subpopulation of 119 women who participated in a validation study of the MoBa FFQ. While data on urinary iodine levels in this sub-sample have previously been reported as 24 h urinary iodine excretion (UIE, μg/24 h), the current study presents these results as urinary iodine concentration (UIC, μg/L). While UIE was relevant for validating estimated iodine intake, UIC is relevant for evaluating iodine status [14]. Details about the study population, urinary sampling and iodine analysis have been described in detail previously [17,18]. Participants in the validation study sub-sample were not representative of all women in MoBa. However, the estimated iodine intake and sources of iodine were similar to the whole cohort [17].

### 2.2. Dietary Information

The MoBa FFQ [23] was specifically planned for the MoBa study and was completed by participating women in gestational weeks 17 to 22. The dietary data used in this study were collected from February 2002 to November 2008. The MoBa FFQ is a semi-quantitative questionnaire designed to capture dietary habits and intake of dietary supplements during the first four to five months of pregnancy and included questions about intake of 255 food items or dishes [22]. For each food item, the frequency of consumption was reported by selecting one out of 8–10 frequencies, ranging from never to several times monthly, weekly or daily. Consumption frequencies were converted into food amounts (g/day) by the use of standard Norwegian portion sizes. Food and nutrient calculations were performed with FoodCalc [24] and the Norwegian food composition table [25]. For calculation of dietary iodine, we used available data from analyses of Norwegian milk and food samples [26,27]. Food items in the FFQ were combined into non-overlapping groups, and the contribution of the various food groups to iodine intake was computed. 

The FFQ did not include any question about the use of table salt or use of iodine fortified table salt. In Norway, few brands of table salt are iodized (maximum 5 μg/g NaCl), and the use of this salt is limited to private households and not allowed in the food industry [27]. Drinking water in Norway contains only negligible amounts of iodine (~2 μg/L) [27]. 

The last page in the FFQ asked about use of food supplements. Thirteen commonly used vitamin, mineral and cod liver oil/fish oil supplements were pre-coded and followed by 6 open-ended spaces where the women were asked to record the name and manufacturer of the supplement(s) used, but not listed. The frequency was reported as one of 9 options of weekly use (never, <1 and 1–7) and the quantity was reported as one of 3 options for liquid supplements and one of 4 options for number(s) of tablets/capsules (1, 2, 3 and ≥4). For calculating nutrients from dietary supplements, an Access database (Microsoft Office 2003) containing the nutrient value of more than 1000 different food supplements was constructed. Commonly sold food supplements in Norway were registered by information provided by the manufacturer, whereas nutritional information of dietary supplements bought from the Internet or abroad were collected from the Internet, either found on the homepage of the manufacturer or the supplier [19]. 

A validation study showed that, relative to a dietary reference method and several biological markers, the MoBa FFQ produces a realistic estimate of habitual intake and is a valid tool for ranking pregnant women according to high and low intakes of energy, nutrients and foods [28]. The relative validity of iodine intake from food and supplements and the intake of specific food groups, such as dairy products and seafood, was evaluated separately. Total iodine intake by the FFQ showed acceptable agreement with iodine intake by the food diary (*r* = 0.48, 95% CI: 0.33, 0.61) and with urinary iodine excretion (*r* = 0.42, 95% CI: 0.26, 0.56). Urinary iodine excretion reflected the important food sources and whether or not iodine was contributed by supplements [17,18,29]. Women were recruited to the validation study between January 2003 and February 2004, and a seasonal difference in urinary iodine excretion reflected a seasonal difference in the iodine content of Norwegian milk [17]. 

The intake of all types of milk and yogurt, except that used in mixed dishes, was divided in several ways: first, into three categories (<200 mL/day, 200–399 mL/day and ≥400 mL/day); second, into six groups (<100 mL/day, 100–199 mL/day, 200–299 mL/day, 300–399 mL/day, 400–499 mL/day and ≥500 mL/day); and third, into two categories (<200 mL/day or ≥200 mL/day). The intake of seafood was divided first into five groups (<5 g/day, 5–19 g/day, 20–39 g/day, 40–59 g/day and ≥60 g/day) and, second, into two categories (<20 g/day or ≥20 g/day). Egg intake was divided into two categories (<8 g/day or ≥8 g/day). Total energy intake was treated as a continuous variable.

### 2.3. Other Variables

Maternal age at delivery was divided into four categories (<25, 25–29, 30–34 and ≥35 years). Marital status was divided into two categories (married/cohabiting or single). Self-reported pre-pregnancy height and weight were used to calculate body mass index (kg/m^2^), which was divided into WHO categories (<18.5, 18.5–24.9, 25–29.9 and ≥30 kg/m^2^), length of education into three categories (≤12, 13–16 or ≥17 years), total household income into three categories (both participant and her partner <NOK 300,000, one ≥NOK 300,000 or both partners ≥NOK 300,000) and pre-pregnant smoking in three categories (non-smokers, occasional smokers or daily smokers). Parity was divided into three categories (nulliparous, primiparous or multiparous). 

### 2.4. Statistical Methods

The estimated intakes of iodine from food and supplements were skewed and are presented by the median and the 5th and 95th percentiles (P5, P95). For univariate analyses, we used the Mann-Whitney *U* test for two group comparisons and the Kruskall-Wallis test for multiple group comparisons of iodine intakes. We used multiple logistic regression analysis to identify predictors of low and suboptimal iodine intake and present crude and adjusted odds ratios (OR) with 95% confidence intervals (CIs). Maternal characteristics and lifestyle variables examined as potential predictors were: maternal age, parity, education, marital status, smoking, pre-pregnancy BMI, household income, iodine containing supplement use and intakes of milk and yogurt, eggs and seafood. As iodine from food increased with increasing food intake, we adjusted for total energy intake. The significance level was set at 5% (two-tailed), and all analyses were performed using the statistical software PASW statistics 17 (SPSS Inc., IBM Company, Chicago, IL, USA).

## 3. Results

The median intake of iodine from food was 141 μg/day, and the median total intake from food and supplements was 166 μg/day. Iodine was obtained from supplements in 19,575 (31.6%) of the women, and the median intake contributed by supplements in this group was 107 μg/day, resulting in a median total intake of 252 μg/day in iodine supplement users. Very low iodine intake (<70 μg/day) was observed for 4.8%, low intake (<100 μg/day) for 16.1% and suboptimal intake (<150 μg/day) was observed for 42% of all participants. The prevalence of low and suboptimal intake was much higher in women who did not obtain iodine from supplements (Table 1). In the total group, 54.3% had iodine intakes <175 μg/day, while the proportion was 70.5% in non-iodine supplement users. In the upper end of iodine intake, two women had intakes above 1100 μg/day, which is considered the upper limit of intake that is unlikely to cause adverse health effects, while 770 women (1.2%) had excessive intakes (≥500 μg/day).

**Table 1:** Iodine intake in 61,904 pregnant Norwegian women in the Norwegian Mother and Child Cohort Study, 2002–2008.

	All			Iodine containing supplement user			
				No		Yes	
*n* (%)	61,904			42,329 (68.4)		19,575 (31.6)	
Iodine intake, μg/day	*n*	%	Cum%	*n*	Cum%	*n*	Cum%
<50	674	1.1	1.1	667	1.6	7	0.0
50–70	2272	3.7	4.8	2208	6.8	64	0.4
70–100	7006	11.3	16.1	6658	22.5	348	2.1
100–150	16,061	25.9	42.0	14,267	56.2	1794	11.3
150–175	7572	12.2	54.3	6044	70.5	1528	19.1
175–250	14,886	24.0	78.3	8970	91.7	5916	49.3
250–500	12,663	20.5	98.8	3463	99.9	9200	96.3
≥500	770	1.2	100	52	100	718	100

Iodine from food did not differ with regard to maternal age, education, use of supplements or marital status, but was lower in smokers than in non-smokers and higher in multiparous than in nulliparous women. Total iodine intake was also higher in nulliparous women than in parous women. Total iodine decreased with increasing BMI, but the negative association was confounded by total energy intake and disappeared when energy was taken into account (data not shown). The most outstanding differences in iodine intakes were found with regards to differences in milk, seafood and egg consumption and whether or not iodine was obtained from supplements (Table 2).

**Table 2:** Iodine intake, median and range (5th percentile, 95th percentile) by maternal characteristics.

			Iodine from food		Total iodine	
		*n* (%)	Median (P5, P95)	*p-*Value *	Median (P5, P95)	*p-*Value *
			μg/day		μg/day	
All		61,904	141 (65, 280)		166 (71, 369)	
*Maternal age*						
	<25 years	6880 (11.1)	141 (61, 305)	0.406	164 (65, 389)	0.207
	25–29 years	20,926 (38.3)	141 (64, 282)		167 (70, 373)	
	30–34 years	23,554 (38.0)	141 (66, 274)		165 (73, 362)	
	≥35 years	10,544 (17.0)	141 (67, 275)		166 (74, 367)	
*Parity*						
	Nulliparous	32,053 (51.8)	139 (64, 280)	<0.001	169 (71, 381)	
	Primiparous	19,262 (31.1)	141 (65, 279)		162 (70, 357)	<0.001
	Multiparous	10,589 (17.1)	147 (67, 284)		164 (72, 356)	
*Maternal education*						
	≤12 years	19,809 (32.0)	140 (60, 295)	0.607	163 (65, 372)	<0.001
	13–16 years	26,612 (43.0)	141 (66, 276)		167 (73, 369)	
	≥17 years	15,484 (25.0)	141 (70, 267)		167 (76, 366)	
*Marital status*						
	Married/cohabiting	59,726 (96.5)	141 (65, 279)	0.083	166 (71, 368)	0.246
	Not married/cohabiting	2178 (3.5)	143 (61, 302)		168 (67, 388)	
*Smoking prior to pregnancy*						
	Non smoker	44,005 (71.1)	142 (66, 277)	0.026	166 (72, 367)	0.015
	Occasional smoker	6376 (10.3)	140 (66, 276)		165 (72, 368)	
	Daily smoker	11,523 (18.6)	139 (61, 294)		164 (66, 377)	
*Pre-pregnancy BMI*						
	<18.5 kg/m^2^	1809 (2.9)	140 (62, 271)	<0.001	168 (71, 376)	<0.001
	18.5–24.9 kg/m^2^	40,457 (65.4)	142 (67, 278)		167 (74, 368)	
	25–29.9 kg/m^2^	13,604 (22.0)	140 (62, 285)		163 (67, 372)	
	≥30 kg/m^2^	6034 (9.7)	137 (58, 288)		160 (63, 372)	
*Income*						
	Both <300,000 NOK	18,929 (30.6)	143 (63, 291)	<0.001	167 (69, 371)	0.073
	One ≥300,000 NOK	26,419 (42.7)	142 (65, 283)		166 (71, 371)	
	Both ≥300,000 NOK	16,556 (26.7)	138 (67, 260)		164 (74, 363)	
*Milk and yogurt intake*						
	<200 mL/day	17,187 (27,8)	88 (49, 141)	<0.001	102 (53, 262)	<0.001
	200–399 mL/day	13,378 (21.6)	122 (84, 175)		137 (88, 296)	
	≥400 mL/day	31,339 (50.6)	183 (123, 320)		209 (129, 410)	
*Fish and seafood intake*						
	<20 g/day	14,195 (22.9)	117 (49, 252)	<0.001	141 (54, 346)	<0.001
	≥20 g/day	47,709 (77.1)	148 (74, 286)		172 (79, 376)	
*Egg intake*						
	<8 g/day	19,921 (32.2)	129 (56, 269)	<0.001	153 (61, 357)	<0.001
	≥8 g/day	41,983 (67.8)	146 (71, 283)		171 (77, 375 )	
*Iodine from supplements*						
	No	42,329 (68.4)	141 (65, 279)	<0.001	141 (65, 279)	<0.001
	Yes	19,575 (31.6)	141 (65, 282)		252 (122, 466)	

* p-Values; unadjusted differences between groups. P5: 5th percentile, P95: 95th percentile.

In women who did not use iodine containing supplements and had low intake of milk/yogurt (<200 mL/day), 66.9% had iodine intake <100 μg/day and 98.6% had intake <150 μg/day. For comparison, in iodine supplement users with low milk/yogurt intake, the corresponding figures were 7.9% and 29.9%. No women had iodine intake <100 μg/day if they obtained iodine from supplements and also consumed at least one daily serving of milk and/or yogurt. 

**Figure 1:**
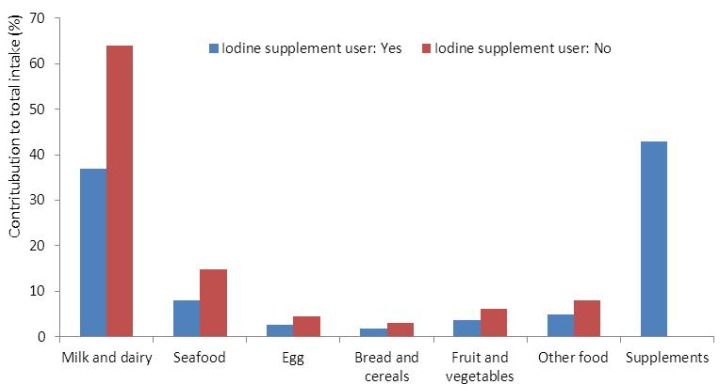
The contribution (%) to maternal iodine intake from food groups and iodine containing supplements in iodine supplement (*n* = 19,575) and non-supplement users (*n* = 42,329).

Iodine from supplements contributed 19% and dairy products contributed 52% to the total iodine intake in the whole group. In women who did not obtain iodine from supplements, dairy products contributed on average 64% and seafood contributed 15% to the total iodine intake (Figure 1).

**Figure 2:**
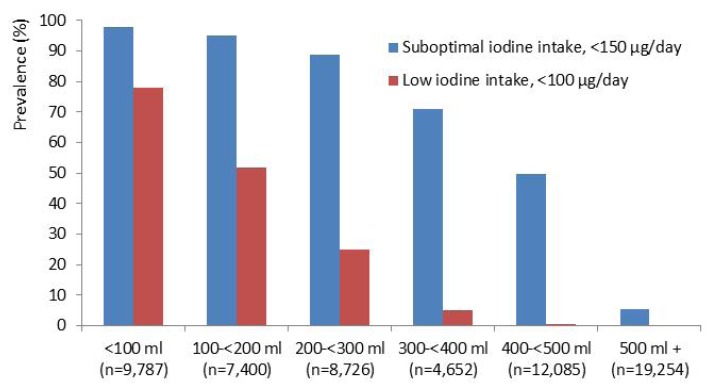
The prevalence of suboptimal (<150 μg/day) and low (<100 μg/day) iodine intake by increasing consumption of milk and/or yogurt in 42,329 non-iodine supplement users.

The prevalence of inadequate iodine intake decreased with increasing milk and yogurt consumption. The results indicate that an average milk/yogurt intake of 200–300 mL/day (1–2 servings) in addition to iodine from other foods, but no iodine from supplements, would secure 100 μg iodine daily for most women, while higher milk and yogurt intake is needed to obtain 150 μg iodine daily (Figure 2).

**Figure 3:**
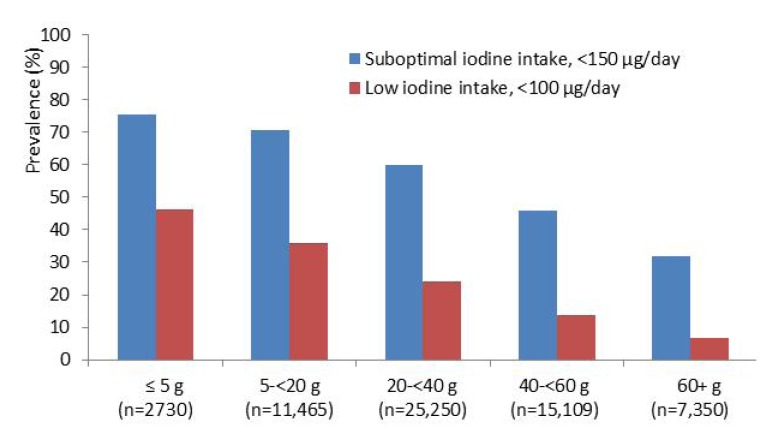
The prevalence of suboptimal (<150 μg/day) and low (<100 μg/day) iodine intake by increasing consumption of seafood in 42,329 non-iodine supplement users.

Likewise, the prevalence of inadequate iodine intake decreased with increasing intake of seafood. The results indicate that an average seafood intake of 20–40 g/day in addition to iodine from other foods, but no iodine from supplements, would secure 100 μg iodine for most women (Figure 3). This amount corresponds to one daily portion of fish or other seafood on bread or 1–2 fish dinners weekly.

In the adjusted analysis, taking all maternal characteristic and relevant dietary practices into account, the most important predictors of suboptimal iodine intake were: no iodine supplement use, low consumption of milk and yogurt and low consumption of seafood (Table 3). These variables were identified as the major predictors of low iodine intake, also when inadequate iodine intake was defined as <150 μg/day (data not shown).

**Table 3:** Predictors of iodine intake <100 μg/day in 61,904 pregnant Norwegian women.

		Iodine < 100 μg/day	Unadjusted model	Adjusted model ^1^
		*n* (%)	OR (95% CI)	OR (95% CI)
*Iodine from supplements*				
	Yes	419 (2.1)	1	1
	No	9533 (22.5)	13.3 (12.0, 14.7)	58.6 (52.0, 66.2)
*Low milk/yoghurt intake*				
	No: ≥200 mL/day	1723 (3.9)	1	1
	Yes: <200 mL/day	8229 (47.9)	22.9 (21.7, 24.3)	42.2 (39.4, 45.7)
*Low fish and seafood intake*				
	No: ≥20 g/day	6093 (12.8)	1	1
	Yes: <20 g/day	3859 (27.2)	2.6 (2.4, 2.7)	3.8 (3.5, 4.1)
*Low egg intake*				
	No: ≥8 g/day	5584 (13.3)	1	1
	Yes: <8 g/day	4368 (21.9)	1.8 (1.7, 1.9)	1.5 (1.4, 1.6)

^1^ Additionally adjusted for maternal age, parity, education, marital status, smoking, body mass index, household income and total energy intake.

To examine the possible change in dietary practices over time, we examined sources and intake of iodine by year of delivery for the women in our study (Table 4). The proportion of women having iodine intake below 100 μg/day increased from 14.8% to 17.1% from 2002 to 2008. There was also an increase in the proportion of women who obtained iodine from supplements. However, there was a 12% reduction in the median consumption of milk and yogurt, from 413 mL/day to 363 mL/day, and a corresponding reduction in iodine contributed by milk and yogurt.

**Table 4:** Intake of iodine and selected iodine sources by year of delivery, *n* = 61,904 pregnant women.

Year	2002	2003	2004	2005	2006	2007	2008
Number of women	3997	10,552	11,020	11,286	11,921	10,246	2882
Iodine <100 μg/day (%)	14.8	15.6	16.2	15.9	15.7	16.3	17.1
Iodine supplement use (%)	26.5	25.8	31.6	34.5	34.7	32.9	31.5
Iodine from food, median μg/day	147	144	141	141	140	138	137
Iodine from milk and yogurt, median μg/day	75	74	72	73	70	68	66
Iodine from other dairy products, median μg/day	14	14	14	14	14	14	14
Iodine contributed by seafood, median μg/day	23	22	21	20	20	19	19
Milk and yogurt, median mL/day	413	409	402	408	400	381	363
Seafood, median g/day	35	34	33	33	33	33	32

**Figure 4:**
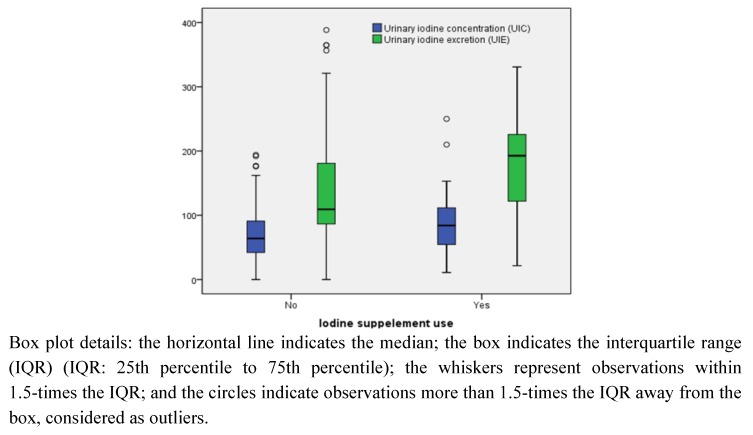
Urinary iodine concentration (UIC, μg/L) and urinary iodine excretion (UIE, μg/24 h) in 119 pregnant women in the Norwegian Mother and Child Cohort Study.

Urinary iodine concentration was measured in 24 h urine samples collected for 119 MoBa participants in 2003–2004. Median UIC was 69 μg/L in the total sample, 64 μg/L in non-iodine supplement users (*n* = 84) and 84 μg/L in iodine supplement users. The corresponding median UIE was 130 μg/24 h in the total sample, 110 μg/24 h in non-iodine supplement users and 190 μg/24 h in iodine supplement users (Figure 4). Only 13 (11%) of the women with available UIC data had UIC ≥ 150 μg/L, while 106 (89%) had UIC < 150 μg/L. The median iodine intake in the 13 women with UIC ≥ 150 μg/L was 150 μg/day, and the median intake in the 106 women with UIC < 150 μg/L was 130 μg/day. However, the WHO reference ranges for defining optimal iodine status based on UIC are for larger population groups [14].

## 4. Discussion

Although there is no screening program for iodine deficiency, Norway has been considered iodine replete for six decades. This study shows that inadequate iodine intake is prevalent in pregnant Norwegian women and suggests that iodine nutrition is a health concern. There are few dietary sources of iodine, and the individual intake of these food groups vary significantly. Pregnant women who do not consume or have low intake of dairy and/or seafood and who do not obtain iodine from supplements are at great risk of having inadequate iodine intake. 

The use of an FFQ to assess iodine intake, as well as assigning an average iodine concentration to all milk and milk products, may result in imprecision in the intake estimates. However, in the present study, we based the estimates mainly on iodine concentrations measured in Norwegian food items and a detailed database for iodine content in specified dietary supplements. Data on urinary iodine excretion in a healthy and highly educated subsample of pregnant women supported that inadequate iodine intake should be regarded as a health concern also in Norway. 

Low iodine intake in women of child-bearing age has been documented in other studies in Norway [27,30] and in other European countries [31,32,33,34,35,36,37]. Assessment of urinary iodine excretion in Western and Central Europe indicated that more than half of the population is at risk of iodine deficiency [2,38]. This is an issue of public concern, especially in regard to pregnant women [4]. A pilot study surveying the prevalence of iodine deficiency in the northeast of England concluded that 3.5% of the pregnant women had evidence of iodine deficiency and that 40% might be borderline deficient, defined by the urinary iodine/creatinine ratio [33]. Insufficient iodine nutrition in pregnant populations have been reported also in the US, New Zealand and Australia [39,40,41].

The effects of severe iodine deficiency during critical periods of brain development are well documented, while less is known about the consequences of milder forms of iodine deficiency [1,4,12,37,42]. Mild iodine deficiency may influence developmental impairment in children. Although limited, a few studies reported associations between prenatal iodine status or suboptimal maternal iodine intake and cognitive function of infants and children up to 18 months [43,44] and with attention deficit hyperactivity disorder (ADHD) symptoms in children [45,46]. A prospective study comprising 692 mother-children pairs in Holland reported an inverse association between maternal iodine status (UIC) and executive function in children at four years of age [47]. The median UIC in the Dutch study population (203 μg/L) did not suggest iodine insufficiency at the group level. In the subsample of women having urinary iodine measurements in our study, median UIC was much lower (69 μg/L). The detailed assessment of diet and supplement use in MoBa, along with an on-going ADHD sub-study involving clinical examination of three-year-old children with ADHD symptoms, represents a unique opportunity to investigate whether mild to moderate deficiency of iodine during pregnancy is associated with the risk of developing ADHD symptoms at three years of age. The MoBa study is especially suited to study potential associations between inadequate maternal iodine intake and ADHD in children, because the children are otherwise well nourished. 

MoBa is a large pregnancy cohort with participants from both urban and rural regions, representing all age groups and all socioeconomic groups. The study group is not entirely representative of the whole pregnant population of Norway, being somewhat better educated and with a lower percentage of smokers than the overall population of pregnant women [48]. The study did not aim to include minority groups, and more than 99% of the participants are of Caucasian ethnicity. Thus, it is all the more remarkable that less than half of the study group had a total iodine intake equal to or above the recommended intake of 175 μg/day, and it is likely that suboptimal iodine intake will be even more prevalent in the total population of pregnant Norwegian women. Data on urinary iodine concentration in the subsample corroborated that inadequate iodine status may be non-trivial even in a privileged pregnancy population in Norway. However, the WHO reference ranges for defining optimal iodine status are based on UIC for population medians [14,49]. The sample of 119 pregnant women is too small to evaluate iodine status in pregnant Norwegians in general, but the results indicate that suboptimal iodine nutrition is likely. Hence, iodine status should be assessed in a larger sample of pregnant women and in school children, as encouraged by WHO [14].

In this study, we combined food frequencies with iodine content measured primarily in Norwegian food samples. The highest iodine content was found in foods of marine origin, with lean fish, such as cod, having more than twice the content of fatty fish, such as farmed salmon [50]. The consumption of fish, especially lean fish, is declining, and more so in women than in men [51]. The current study showed that milk intake declined over the study period, resulting in a decline in iodine contributed by milk. The iodine content in milk differs with time of year and farming practice [26]. In Norway, low-fat milk from the summer season had significantly lower median iodine concentration (88 μg/L, range 63–122 μg/L) compared with low-fat milk from the winter season (232 μg/L, range 103–272 μg/L). The median iodine concentration of organic summer milk (60 μg/L) was significantly lower than the iodine concentration of organic winter milk (127 μg/L) [26]. A limitation of the present study was that a single average iodine value was applied to all milk and yogurt (150 μg/L). Changes in farming practices and legislation may have resulted in lower iodine content in milk over the last decade, but no values of iodine content in Norwegian milk have been published since 2003.

Estimating iodine intake using dietary assessment methods has limitations and uncertainties. The contribution of iodine from iodine fortified household salt was not included in the current study. However, the fortified salt contains very little iodine (5 μg/g), and the use of table salt is low (<2 g/day). Other concerns related to estimating iodine intake are recall bias and changes in appetite and eating patterns due to pregnancy. Misreporting is a serious error in all dietary assessment, and recall of diet over the first trimester is particularly difficult, as many women experience nausea and changes in appetite and eating patterns. Results from the validation study showed that stronger agreement between the FFQ and the test methods was observed for foods perceived as “healthy” than for “unhealthy” foods. Likewise, stronger agreement was seen for total energy intake when we excluded women who reported nausea at the time of answering the FFQ [28]. It should also be noted that in this descriptive study, we used the MoBa FFQ to identify risk groups of low iodine intake, as well as to evaluate dietary sources of iodine, and these data are not independent. It is difficult to predict whether uncertainties in the dietary assessment would most likely lead to over- or under-estimating iodine intake. The results from the subsample analysis indicate that iodine intake of at least 150 μg/day would be needed to get the median UIC up to the optimal range of 150–249 μg/L defined by WHO.

The results of the present study highlight the importance of a balanced diet, including milk and seafood, during pregnancy. The significance of these foods as iodine sources has also been reported in other Nordic countries [52,53,54]. Women with low intake of dairy and seafood are particularly vulnerable to low iodine intake and should be encouraged to use iodine-containing dietary supplements. Iodine fortification of table salt is a common strategy for iodine prophylaxis, but this does not ensure sufficient iodine intake in all population groups, except when iodine fortified salt, at concentrations higher than the 5 μg/g NaCl presently permitted in Norway, also is used in bread and other food products [1]. Denmark implemented mandatory iodine fortification of household salt and bread salt at a level of 13 μg/g NaCl (13 ppm) in the year 2000. The Danish iodine fortification program was monitored with regard to positive, as well as negative, health effects. Evaluation of urinary iodine excretion in subgroups of the population before and after fortification showed that fortification resulted in increased iodine intake in all investigated groups [53]. Milk was the strongest dietary determinant of iodine intake before, as well as after, the fortification, and subjects with low milk intake combined with low intake of bread or table salt had iodine intake below the recommended intake, also after fortification.

The importance of iodine-containing supplements has been demonstrated in studies using urinary iodine excretion for assessing sufficient iodine nutrition in pregnancy [1,55]. In Norway, the dietary recommendations to pregnant women do not include use of iodine supplementation. The present study shows that use of iodine-containing supplements is vital to secure optimal iodine intake and is especially important for women who do not include or have low intakes of seafood and/or milk and dairy in their diet. 

## 5. Conclusions

In conclusion, this study shows that the current iodine intake in a large proportion of pregnant Norwegian women may give rise to concern. Although the evidence of deleterious effects in terms of subtle cognitive impairment is limited, it is essential to increase the public awareness of dietary iodine nutrition in pregnant women. Our results highlight the significance of a balanced diet, including milk and seafood, during pregnancy. Women with low intake of dairy and seafood are particularly vulnerable to low iodine intake and should be encouraged to use iodine-containing supplements. The dietary sources of iodine in Norway do not secure a sufficient iodine intake for the entire population, and more awareness on iodine nutrition is warranted, especially in women who, for various reasons, limit their consumption of dairy and seafood. It cannot be excluded that a number of children growing up in Norway today have lifelong impairments due to suboptimal iodine nutrition in early life. There is an urgent need for public health strategies to monitor and secure the iodine status in Norway.
